# Environmental Drivers of Malignant Clonal Selection in Precancerous Lesion Evolution

**DOI:** 10.1111/cpr.70011

**Published:** 2025-02-25

**Authors:** Yancheng Lai, Shaosen Zhang

**Affiliations:** ^1^ Department of Etiology and Carcinogenesis, National Cancer Center/National Clinical Research Center/Cancer Hospital Chinese Academy of Medical Sciences (CAMS) and Peking Union Medical College (PUMC) Beijing China; ^2^ Key Laboratory of Cancer Genomic Biology Chinese Academy of Medical Sciences and Peking Union Medical College Beijing China

## Abstract

Somatic mutations accumulation and subsequent malignant clonal selection are the processes that lead to cancer. The intricacy includes exposure to carcinogens as well as the ways in which these exposures interact with genetic, polygenic, or epigenetic predispositions. It is worthwhile to investigate how environmental factors influence the initial transformation of healthy cells into malignant ones, or their effects on promoting growth, invasion, immune evasion, inflammation and drug resistance of onset cancerous cells.

**Abbreviations**
CAFs: cancer‐associated fibroblasts
CSCs: cancer stem cells
ecDNA: extrachromosomal DNA
ECM: extracellular matrix
FAP: familial adenomatous polyposis
HTAN: Human Tumor Atlas Network
PME: precancer microenvironment

## Main

Tumorigenesis is primarily driven by the accumulation of genetic and epigenetic alterations in oncogenes and tumour suppressor genes [1]. It has been noted that abnormal clones caused by mutations may already be present during the embryonic or infantile period [2], and these clones can be roughly divided into two main categories, the pro‐tumorigenic and the anti‐tumorigenic subtypes. Among the oncogenic clones, the division and expansion of stem cells lead to uncontrolled cell growth and malignant transformation, which is called cancer stem cells (CSCs) [3]. CSCs have been found to possess a multitude of functional properties, including tumour initiation, self‐renewal, multipotency, plasticity, treatment resistance and the ability to maintain cancer stemness as a dynamic cellular state [4]. Despite the accumulation of numerous oncogenic mutations in the normal tissues of healthy individuals as they age, tumour formation remains a rare occurrence [5, 6]. Scientists have discovered that ‘benign clones’ can be found in mutant polyclones to prevent precancerous clones from growing and progressing, but they are then overcome by tumour‐related clones. Finally, nearly all nearby clones that obstruct malignant cloning will eventually be eliminated, giving the local environment an oligoclonal or monoclonal composition [7, 8]. This raises the question: what are the mechanisms that govern the dynamic competition between mutant clones, and how does this competition ultimately lead to premalignant transformation and early tumorigenesis?

To investigate these processes, researchers have initiated efforts to map the clonal evolution and competition throughout the normal‐precancer‐cancer continuum [9, 10]. This involves the integration of spatial and temporal dimensions to illustrate the cellular and molecular evolution from normal tissue through the pre‐cancerous stage to cancer. Unravelling the complex ecosystem of diverse cell types involved in tumorigenesis necessitates more precise assessments of tissue morphology, cellular coordination and intercellular connections. Such insights are crucial for identifying the key drivers of tumorigenesis, their interconnections, and causality, as well as for pinpointing potential therapeutic targets and molecular markers for the early detection of precancerous lesions.

**FIGURE 1:**
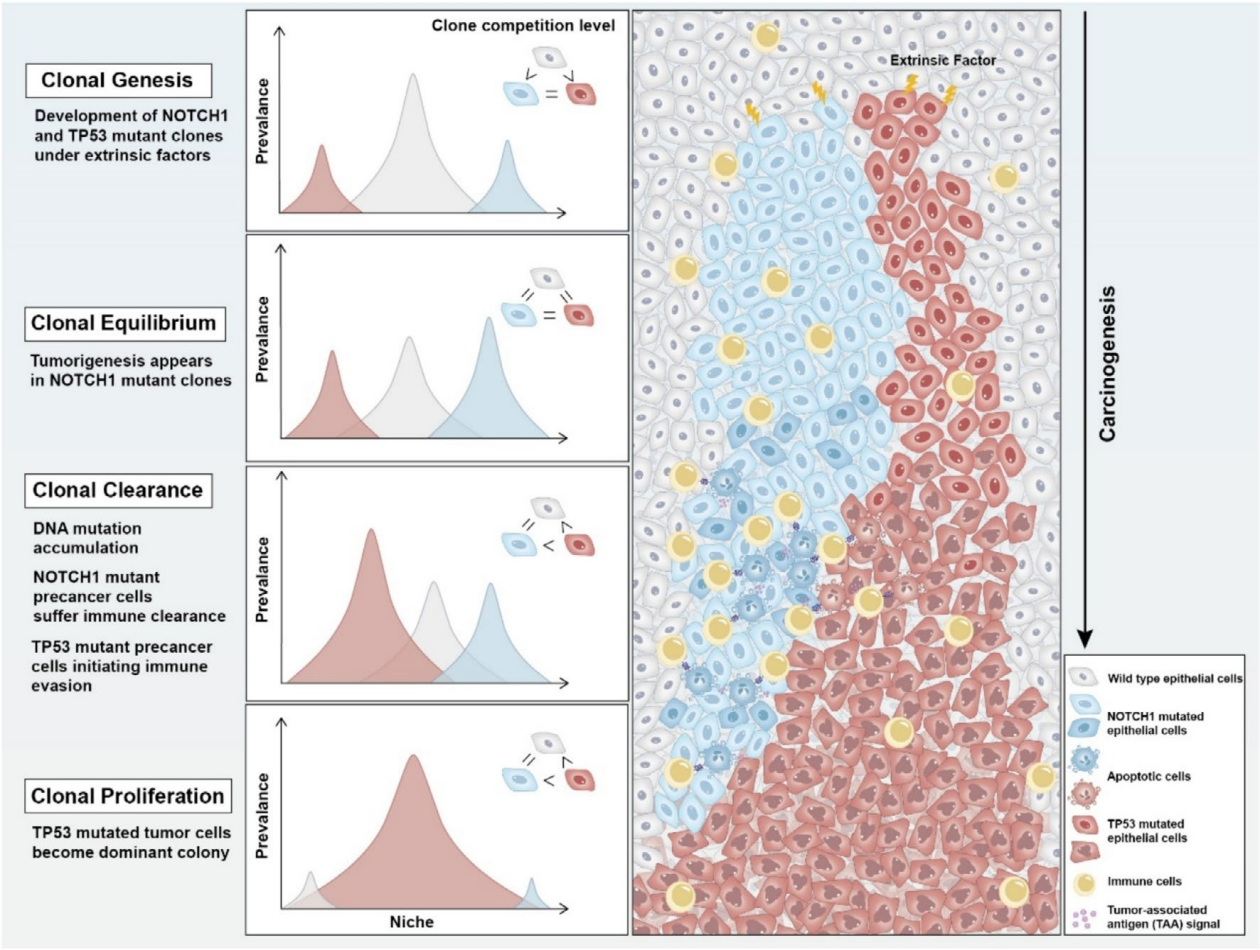
Clonal evolution events during precancerous progression through NOTCH1 and TP53 mutant clones as examples. Four distinct types of clonal events are commonly observed during the progression of precancerous lesions: Clonal Genesis, Clonal Equilibrium, Clonal Clearance and Clonal Proliferation. In the early stages, tumour‐suppressive mutant clones, exemplified by NOTCH1, establish dominance within the local niche and inhibit the expansion of malignant proliferative clones, such as those characterised by TP53 mutations. ‐Along malignant transformation and tumor evolution, NOTCH1‐mutant clones undergo clonal clearance mediated by immune cells, which simultaneously release tumour‐associated antigen (TAA) signals. This process results in the clearance of TAA‐sharing clones, predominantly tumour‐suppressive clones like NOTCH1, while malignant clones such as those with TP53 mutations evade immune recognition and progressively occupy the ecological niche.

Driver mutations may confer advantages that enhance the adaptability and fitness of proliferating cells, leading to a dynamic ecosystem where the most fit clones proliferate, effectively transforming the tissue into a patchwork of mutant populations. For example, the competition between *TP53* and *NOTCH1* mutant clones in oesophageal precancer lesion is intricate and long‐lasting (Figure 1). We observe that the outcomes of this clonal selection appear to be linked to the influence of environmental selection, since immune surveillance and tumour‐associated antigen (TAA) signalling might mediate the removal of *NOTCH1* mutations from the prcancerous lesion, assisting the *TP53* mutant clone in becoming the dominant clone. The destabilisation of early clonal competitive balance, in our opinion, is mainly due to this environment‐induced clonal selection pressure. Colom et al. also demonstrated that early and late lesions in the oesophageal epithelium are subject to distinct clonal selection pressures [11]. Their further study focused on oesophageal cancer models subjected to mutagen exposure and natural aging processes. The authors highlight that cells in the clone centre exhibit genetic homogeneity and lack a selective advantage over adjacent cells. Mutant clone formation predominantly occurs at clone peripheries, where progenitor cells may encounter less fit neighbours. The biological basis underlying a cell's response to the genotype and fitness of its neighbouring cells potentially encompasses cytoskeletal and metabolic pathways, as well as cell–cell communication mechanisms, such as NOTCH signalling. These findings underscore the critical role of the cellular microenvironment in clonal expansion and selection [12].

Recent studies published in the Human Tumor Atlas Network (HTAN) collection of Nature offer novel insights into the evolution of precancerous clones [7, 13, 14, 15]. The primary focus of most investigations has been on changes occurring within or between tumour clones, rather than on external pressures. Despite this, the role of environmental stress in clonal selection is gaining increasing attention. Environmental stress plays a dual role in clonal proliferation and selection. On one hand, it can facilitate cellular survival and acclimation to multifaceted microenvironments. On the other hand, adverse environmental conditions induce clonal competition, which enables malignant clones to emerge as the dominant population. A study has suggested that an immunosuppressive precancer microenvironment (PME) may contribute to monoclonal transformation. Cell‐to‐cell communication analyses based on ligand‐receptor interactions reveal that extracellular matrix (ECM) tissue and cell adhesion mechanisms may underlie the substantially elevated ligand‐receptor interactions between the epithelial subtypes of early polyclonal lesions compared to normal colon and advanced monoclonal lesions [7]. An earlier study by Reeves et al. also demonstrated that during the development of squamous carcinoma, adjacent wild‐type epithelial cells are recruited by the initial clones harbouring *Hras* mutations [16]. Additionally, Esplin et al. conducted a multi‐omics analysis of benign polyps, dysplastic polyps and normal mucosa in familial adenomatous polyposis (FAP) patients, revealing that the transition from precancerous lesions to cancer development entails a dynamic interplay of myriad molecular and cellular processes. These events encompass the immune system, cell division, hormonal signalling, extracellular matrix proteins and metabolic shifts, including alterations in lipid and amino acid metabolism [17]. The results of the article also indicate that connections between the cell types involved in 63 proteomic and transcriptomic immunity pathways, which were enriched along the progression from normal to undesirable lesions, also revealed an extensive network of immune and neuronal cell types recruited in the Mucosa‐Dysplastic outcome. Additionally, the function of tissue structure and hormone‐mediated estrus cycles in breast duct clonal selection has recently come to light [18]. Taken together, these findings appear to suggest a direct relationship between environmental stress and the emergence of tumour cloning.

## The Future Prospect

Although the clonal evolutionary mechanisms underlying the transition from normal tissue to cancer are being gradually unravelled, it remains elusive how environmental pressures select polyclones in the early stages of tumorigenesis and ultimately drive their progression into malignant monoclones. Several ‘benign clones’ reside in normal human tissues, and when exposed to stressors such as environmental stimulation, these clones evolve into one or a limited number of ‘malignant clones’. This process involves clonal evolution and competition, alterations in the composition and properties of the milieu, synergistic molecular pathways between epithelial cells and the microenvironment, and significant external environment‐driven events. We posit that this is a crucial question that has been overlooked by the majority of previous studies.

One intriguing hypothesis is that the robust cellular communication exhibited by early tumour polyclones may be a result of clones communicating more with each other in an attempt to find ‘allies’, an effect that is attenuated in the late stable monoclonal environment [7]. During this process, the PME undergoes dynamic evolution, with the pattern of cellular interactions gradually shifting from benign to one that facilitates cancer progression. This transition can be attributed to various factors, including external photoelectric signals, hypoxia, alterations in pH and changes in the composition of the microbiome. Notably, the involvement of early immune response cells, such as neutrophils and antigen‐presenting cells, in clonal competition for precancer lesions may have more diverse functions than previously thought. While it has been shown that these factors can modify the microenvironment to create more aggressive and proliferative tumours, further investigation is necessary to elucidate the mechanisms by which clonal selection is linked to these factors.

Environmental stress plays a significant role in clonal selection, particularly during the transition from precancerous lesions to malignancy. Future research should investigate common characteristics of precancerous clonal selection across a broader spectrum of cancer lineages, as previous studies have focused on major tumour types such as breast, lung and colorectal cancer. Furthermore, to better understand the complex interactions and selection dynamics that occur within clones and between clones and microenvironments during the multistage evolution of normal‐precancer‐cancer, we need to establish a novel research paradigm that integrates spatiotemporal multiomics analysis, artificial intelligence‐driven computational strategies and advanced functional validation techniques.

Specifically, research relies on the accurate and efficient collection of human tissue samples through endoscopy and other screening techniques, as well as the establishment of a cohort that spans various stages of cancer development. In this context, the application of spatiotemporal multi‐omics analysis technology will overcome the limitations of traditional single‐cell sequencing and restore the fine spatial picture of cell interactions at single‐cell resolution. For instance, by employing methods such as SHARE‐seq [19] and CITE‐seq [20] in conjunction with multi‐stage sample collection, it is possible to achieve the integration of RNA, protein and chromatin information across multiple temporal dimensions. Moreover, the Perturb‐map technology [21] enables the elucidation of functional genomics at single‐cell resolution in the spatial domain. These techniques are crucial for constructing a multidimensional map of cancer initiation and tumour evolution [14].

Furthermore, artificial Intelligence techniques such as machine learning will be essential in integrating heterogeneous data, enhancing biological interpretation and predicting key driving events. For instance, deep learning algorithms like convolutional neural networks (CNNs) and recurrent neural networks (RNNs) can help analyse and interpret complex omics data, while natural language processing can aid in extracting insights from unstructured textual data such as scientific literature and clinical notes. The massive amounts of spatiotemporal omics data pose unprecedented challenges to data mining. Additional functional verification is required to elucidate the causative involvement of key molecular processes in carcinogenesis. Emerging research platforms, such as organoids and visual animal models, provide powerful tools for multi‐level validation from in vitro to in vivo.

**FIGURE 2:**
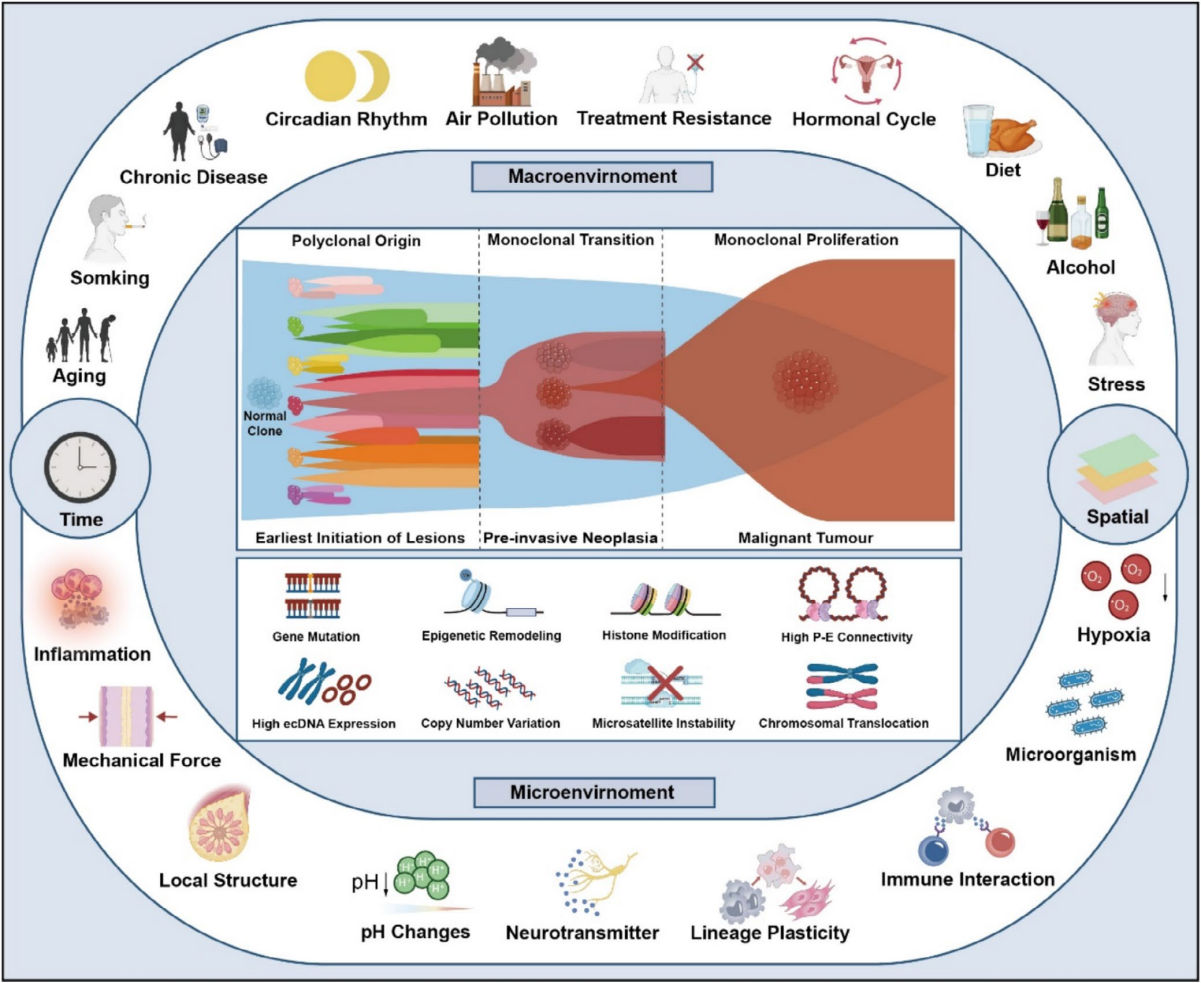
Interplay between environmental drivers and genetic or epigenetic predispositions on polyclonal to malignant clonal transition in precancerous lesion evolution. Somatic mutations accumulation is essential for cancer initiation and development, may arise as a result of spontaneous errors in normal DNA replication, during aging of quiescent cells, or by exposure to mutagens in the environment. Environmental pressures, encompassing both macroenvironmental and microenvironmental factors, play a crucial role in clonal selection and tumour progression across different temporal and spatial dimensions. Carcinogens, like stress, chronic disease, obesity, dietary factors and inflammation may also contribute to mutation burden indirectly. Oncogenic mutations may remain dormant in tissues of healthy individuals for long periods unless the tissue is repeatedly exposed to environmental carcinogens, causing positive selection of malignant cells carrying driver mutations. Known or suspected macro/microenvironmental promoting factors are shown as examples.

Generally speaking, a crucial focus of future cancer research will be the integration of advanced spatiotemporal omics measurements, artificial intelligence analysis, and functional validation techniques to construct multidimensional evolutionary maps of various cancer stages, types and sites. This will necessitate the incorporation of environmental stress, precancerous lesion evolution, clonal selection and microenvironment shaping into a unified research framework. More importantly, we should not disregard or exclude the potential of any newly identified or previously recognised environmental factors or mechanisms in driving alterations in cellular behaviour that influence clonal selection. These factors encompass the recently elucidated mechanisms of microenvironmental mitochondrial transfer [22], as well as mechanical stress [23] and peritumoral neurotransmitters [24] (Figure 2). Moreover, we should also focus on the stem‐like cell populations within these aberrant clones and investigate how environmental factors influence their behaviour and dynamics [25, 26]. Overall, understanding and grasping the temporal and spatial nodes of macroscopic and microscopic environmental pressures in the polyclonal to monoclonal selection of normal‐precancerous cancers, along with the molecular mechanisms, will provide key insights to elucidate the general features of tumorigenesis. This will also establish the foundation for the development of personalised early prevention and treatment strategies.

## Author Contributions

S.Z. conceptualised and supervised the study. Y.L. drafted the manuscript and prepared the figures. S.Z. and Y.L. reviewed and prepared the final manuscript.

## Conflicts of Interest

The authors declare no conflicts of interest.

## Data Availability

Data sharing is not applicable to this article as no new data were created or analyzed in this study.
